# Endocrine regulation of circadian rhythms

**DOI:** 10.1038/s44323-025-00024-6

**Published:** 2025-03-08

**Authors:** Kimberly Begemann, Oliver Rawashdeh, Iwona Olejniczak, Violetta Pilorz, Leonardo Vinícius Monteiro de Assis, Jazmin Osorio-Mendoza, Henrik Oster

**Affiliations:** 1 Institute of Neurobiology, University of Lübeck, Lübeck, Germany; 2 Center of Brain, Behavior, and Metabolism, University of Lübeck, Lübeck, Germany; 3 School of Biomedical Sciences, Faculty of Medicine, The University of Queensland, Brisbane, QLD Australia

**Keywords:** Hormones, Circadian regulation, Circadian mechanisms

## Abstract

Circadian clocks are internal timekeepers enabling organisms to adapt to recurrent events in their environment – such as the succession of day and night—by controlling essential behaviors such as food intake or the sleep-wake cycle. A ubiquitous cellular clock network regulates numerous physiological processes including the endocrine system. Levels of several hormones such as melatonin, cortisol, sex hormones, thyroid stimulating hormone as well as a number of metabolic factors vary across the day, and some of them, in turn, can feedback on circadian clock rhythms. In this review, we dissect the principal ways by which hormones can regulate circadian rhythms in target tissues – as phasic drivers of physiological rhythms, as *zeitgebers* resetting tissue clock phase, or as tuners, affecting downstream rhythms in a more tonic fashion without affecting the core clock. These data emphasize the intricate interaction of the endocrine system and circadian rhythms and offer inroads into tissue-specific manipulation of circadian organization.

## Introduction

Most organisms are exposed to recurring changes in environmental conditions and express physiological rhythms over the 24-h day. To synchronize the organism to these predictable rhythms, circadian clocks (Latin “circa diem” meaning “about a day”) have evolved. On the molecular level, clock genes and their protein products oscillate to generate these circadian rhythms. In mammals, heterodimers of Brain and muscle ARNT-like protein-1 (BMAL1) and Circadian locomotor output cycles kaput (CLOCK) activate the transcription of the clock genes *Period* (*Per1-3*) and *Cryptochrome* (*Cry1/2*) throughout the day while their protein products, PER1-3 and CRY1/2, inhibit CLOCK:BMAL1 and consequently their own transcription during the night^1^. This core transcriptional-translational feedback loop is stabilized by additional auxiliary loops^1^.

The molecular clock machinery is found in most cells and, thus, for coherent timing, different tissue clocks are synchronized by a master pacemaker located in the suprachiasmatic nucleus (SCN) of the hypothalamus^1,2^. Receiving light information via intrinsically photosensitive retinal ganglion cells and the retinohypothalamic tract, SCN neuronal activity synchronizes to the external light-dark cycle^2^. The SCN communicates with extra-SCN clocks in the brain and peripheral tissue clocks to regulate neuronal, behavioral, humoral, and physiological functions^3^. While rhythmic light input is not required to elicit rhythmicity in the SCN itself, coordination of peripheral tissue clocks requires a rhythmic environment and the SCN becomes crucial for overall rhythmicity under non-rhythmic conflicting *zeitgeber* (German: “time givers”) conditions^4,5^. Non-photic *zeitgebers* such as feeding-fasting signals or external temperature rhythms can reset tissue clocks independent of the SCN^6^. Additionally, there are endogenous time signals such as body temperature rhythms or rhythms in hormonal or neuronal activity affecting the circadian clock network^7,8^.

**Fig. 1:**
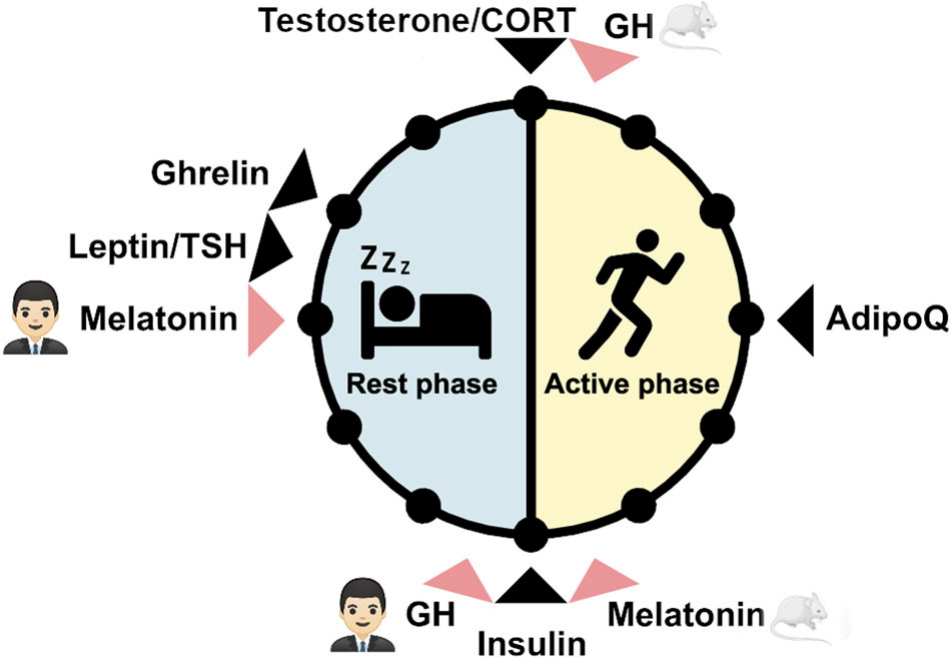
Peak times of hormones during the rest and active phase in humans and nocturnal rodents. The time window of highest hormone levels in humans and nocturnal rodents is indicated by a black arrow. Hormones with differences in peak time between humans and nocturnal rodents are indicated with a red arrow. AdipoQ: adiponectin, CORT: cortisol in humans and corticosterone in mice, GH: Growth hormone, TSH: Thyroid stimulating hormone. Mouse image: smart.servier.com.

**Fig. 2:**
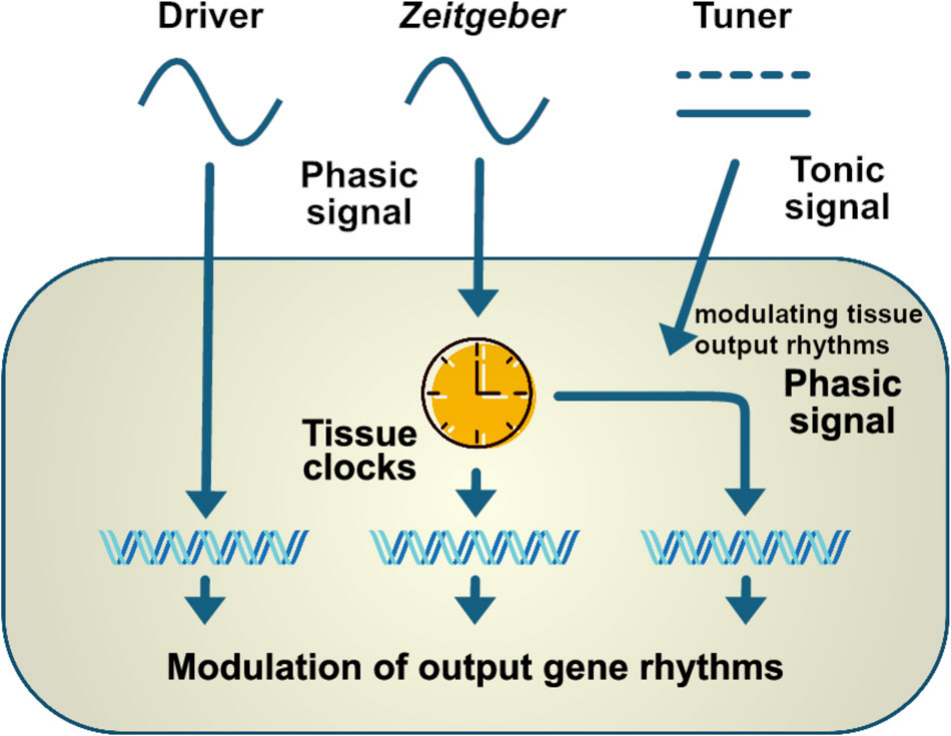
Concept of endocrine regulation of circadian rhythms. Hormones can act as rhythm drivers, *zeitgebers*, or tuners. The function as rhythm driver is clock-independent and requires a rhythmic hormone that can influence rhythmic gene expression via direct hormone-target interactions. As a *zeitgeber*, hormones directly regulate clock gene expression in target tissues and can shift the phase of the clock. As a tuner, the hormonal signal is tonic but able to modulate the rhythmic reception and response to other external stimuli in the target tissue. In this way, a tonic action on the target tissue can modulate gene expression rhythms, thus eliciting a phasic response.

Many hormones are known to oscillate throughout the 24-h day including melatonin, glucocorticoids, sex steroids, thyroid stimulating hormone, and several metabolic hormones such as adiponectin, leptin, ghrelin, insulin, and glucagon (Fig. 1)^9–17^. Importantly, metabolic hormone rhythms are also influenced by external stimuli such as the timing of nutrient uptake^6^. Oscillations of some hormones depend on sleep or sleep stage. Renin, for example, oscillates during sleep with lower levels during rapid eye movement (REM) sleep and higher levels during non-REM sleep^18^. Growth hormone (GH) secretion peaks during sleep initiation and is positively correlated with renin levels^19,20^. In this review, we conceptualize how these hormones can regulate circadian rhythms in different tissues (Fig. 2). First of all, hormones can be rhythm drivers, i.e., the hormone itself is rhythmic and thereby regulates the rhythmic expression of other genes controlling physiological functions. This regulation is target tissue clock-independent and instead archieved by direct hormone-target interaction. Glucocorticoids, for example, regulate the expression of glucocorticoid-sensitive genes by binding to glucocorticoid (GR) or mineralocorticoid receptors (MR) and subsequent activation of transcription through glucocorticoid/mineralocorticoid response elements (*GREs*/*MREs*) in gene regulatory regions^21,22^. However, several clock genes also contain glucocorticoid response elements^23^. Consequently, glucocorticoids are at the same time rhythm drivers for these respective clock genes, thus affecting tissue clock regulation (and downstream functions), i.e., they act as *zeitgebers*. Similarly, melatonin or insulin can affect tissue clock gene expression, thereby resetting local circadian clocks^24,25^. The third possibility of endocrine regulation of circadian rhythms is termed “tuning”, a concept that we recently suggested for thyroid hormones in the liver^26^. In this case, a largely arrhythmic hormonal signal triggers a rhythmic reception and response in the target tissue, thus changing tissue output rhythms, albeit without affecting core clock rhythms, in response to alterations in endocrine signal tone (or level). Independent of this, hormone action can additionally be affected by circadian gating. In this case, the local clock determines when a tissue is more or less sensitive to respond to hormonal stimulation. Well-known examples are the adrenal clock that gates glucocorticoid release in response to adrenocorticotropin (ACTH) stimulation or glucose-stimulated insulin secretion in the pancreas^10,27^.

## Melatonin

Melatonin is a hormone that plays a crucial role in regulating circadian rhythms. It acts as a direct circadian rhythm driver and a *zeitgeber*, exerting significant influence on various physiological processes^28^. Produced primarily by the pineal gland, melatonin secretion is intricately regulated by the light-dark cycle, with levels rising in the evening and peaking during the night in humans to time sleep onset by reducing wakefulness, then declining in the early morning, which facilitates wakefulness. In nocturnal rodents, melatonin peaks in the end of the active phase. This rhythmic production and secretion of melatonin is driven by the SCN clock in the hypothalamus which integrates light signals via the retinohypothalamic tract to synchronize internal biological clocks with the external environment. Collectively, this is achieved by the SCN transmitting two types of regulatory signals to the pineal. One is a signal that is coupled to the SCN circadian clock which restricts melatonin synthesis and release to the nocturnal phase of the circadian cycle in humans, and the second is an inhibitory signal, transmitting incidental nighttime light exposure to acutely interrupt melatonin synthesis and release^29,30^.

Evidence suggests that melatonin can act on circadian rhythms by directly influencing the activity of the SCN by both acute and clock-resetting mechanisms. Melatonin’s daily action on the SCN physiology helps orchestrate the timing and synchronization of various biological rhythms such as sleep-wake cycles, hormone secretion, and core body temperature fluctuations^31^. As such, exogenous melatonin can help entrain, or synchronize, circadian rhythms in individuals with disrupted sleep patterns, such as shift workers or those suffering from jet lag, by re-establishing the correct phase alignment of the internal clock and thereby restoring normal circadian rhythms^32^. The interaction of melatonin with the SCN is mediated by melatonin receptor signaling, which helps regulate its daily oscillations and thus ensuring that downstream biological processes maintain a consistent ~24-h rhythm.

As a *zeitgeber*, melatonin serves as an internal cue that helps synchronize the body’s internal clocks with external time cues, particularly in low-light or dark conditions where external cues might be weak or absent^33^. For instance, timed melatonin intake can advance or delay circadian phases, helping to manage conditions like delayed sleep phase disorder and aiding in the adaptation to new time zones^34,35^.

Melatonin refines the amplitude and robustness of circadian rhythms. It influences the activity and modulates the response of retinal cells to light. By signaling via the melatonin receptor 2 (MT2), melatonin influences the activity of retinal ganglion cells and other retinal neurons, which helps regulate the intensity and quality of the light signals transmitted to the SCN. Pineal melatonin can also directly modulate the sensitivity of the SCN to *zeitgebers*, thereby influencing the overall stability and adaptability of the circadian system. The two melatonin receptors (MT1 and 2) are found in various tissues and organs^36^. G-protein coupled receptor binding of melatonin activates different signal cascades that can also affect gene transcription^37^. Through these receptors, melatonin can affect various peripheral clocks, ensuring that local rhythms are in harmony with the central clock in the SCN. This coordination is crucial for optimal physiological functioning, as misalignment between different body clocks can lead to metabolic, cardiovascular, and psychological disorders^38^. The hormone’s influence on mood disorders highlights its broader significance in maintaining mental health, as disruptions in circadian rhythms as well as a dysregulation in melatonin secretion are often linked to mood spectrum disorders, including bipolar disorder, major depressive disorder, and seasonal affective disorder^39–41^.

## Glucocorticoids

Glucocorticoids (GCs) are steroid hormones produced by the zona fasciculata of the adrenal cortex. They affect many physiological processes, most notably metabolism and the immune system^42^. Under baseline conditions, they are produced in a circadian manner, with the peak occurring shortly before, or in anticipation of the active phase (dawn for diurnal, dusk for nocturnal animals). Superimposed on this circadian rhythm is an ultradian rhythm of release with peaks occurring approximately every 90 min, although they are more variable in frequency and amplitude^43^. The highest pulse of GC release occurs around wake-up time and has thus been termed the cortisol awakening response^44^.

Three separate mechanisms contribute to rhythmic glucocorticoid secretion. Firstly, the hypothalamus-pituitary adrenal (HPA) axis is under circadian control via arginine-vasopressin (AVP) projection from the SCN to the paraventricular nucleus (PVN), generating a rhythmic firing pattern in the downstream region^45^. The PVN releases corticotropin release hormone and AVP into the median eminence, where it can stimulate the corticotropes of the pituitary to release ACTH into the bloodstream. In the adrenal cortex, binding of ACTH by melanocortin 2 receptors initiates the production and release of GCs (cortisol in humans, and corticosterone in rodents). While the entire HPA axis is under circadian control, circulating ACTH levels, show much lower amplitudes than cortisol^44^; therefore, the strong GC rhythm cannot be fully explained by ACTH stimulation. The adrenal also receives innervation from the autonomous nervous system via the splanchnic nerve. This connection has been shown to transmit light information from the SCN directly to the adrenal gland and is responsible for modulating the adrenal sensitivity to ACTH^46–48^. Finally, it has been demonstrated that the adrenal cortex expresses a functional circadian clock, which gates the organ’s sensitivity to ACTH and further contributes to the generation of a robust GC rhythm^10,49^.

Once released into the bloodstream, GCs exert their effect via interaction with two types of nuclear receptors: MR and GR. While MR has a much higher affinity to GCs than GR and is at full occupancy at most times of the day, GR mediates more phasic GC effects. It binds *GREs* to drive transcriptional changes in non-clock as well as clock genes^21,50^. GCs are therefore rhythm drivers regulating rhythmic gene expression via *GREs*, but also *zeitgebers* for peripheral clocks by their action on *Per* expression. Their role as *zeitgeber* is most evident in their function as stress hormones. GCs rise in response to stress, reaching levels above the circadian peak. Stress leads to changes in *Per1* and *Per2* expression and phase shifts of peripheral clocks in a number of tissues, including lung, liver, kidney and multiple brain regions, but excluding the SCN^51^. Exogenous GCs can induce clock gene expression in vivo and are commonly used to synchronize cells in vitro^9,52,53^. The SCN remains isolated from the synchronizing activity of the glucocorticoids, a phenomenon which has historically been attributed to a lack of GR expression in this brain region^9^. Recently, however, GR expression was detected in astrocytes of the adult SCN, suggesting that GC effects on the SCN may be more direct than previously thought^54^.

Another common way to study GC function in the clock and gene expression involves adrenalectomy (ADX). Removing the adrenal gland, and therefore depleting GCs, can affect peripheral tissue clocks, including up- and down-regulation of tissue-specific genes^55^. Interestingly, in ADX rats many peripheral clocks entrain faster to jet-lag than in sham-operated animals^56^. GCs exert a stabilizing function on liver peripheral clock rhythms, with the liver clock adjusting to a temporally restricted feeding schedule faster in ADX mice^57^. These results are the basis of a theory that GCs protect the peripheral rhythms from external *zeitgebers* and transient disturbances. It is therefore of concern that our increased exposure to artificial light at night can both suppress and phase advance GC rhythms^58^, potentially rendering us more susceptible to circadian disruption.

GCs have complex effects on food intake and energy metabolism, specifically underlined by the diverging effects under acute and chronic stress. It has catabolic effects on energy stores such as adipose tissue and muscle, with the goal of mobilizing glucose into the blood to sustain the brain in the fight-or-flight response. However, when stress becomes chronic, the anabolic effects of this hormone start to prevail. In Cushing’s syndrome, for example, chronically high GC levels promote insulin resistance and central fat accumulation^59^. GCs further increase the preference for palatable, high-caloric foods which predisposes to overconsumption and obesity^60^.

The relationship between GCs, food intake and appetite is reciprocal, as disrupted feeding schedules shift the daily GC rhythm, for example in rodent daytime feeding paradigms^61,62^, while ADX animals with phase-shifted GC supplementation consume almost half of their calories in the inactive phase^63^. In addition, orexigenic and anorexigenic neuropeptides are dysregulated under anti-phasic GC supplementation, with NPY expression under direct GC/GR transcriptional control phase^63^.

GC interactions with metabolic hormones are complex and have been reviewed elsewhere^60,64,65^. Briefly, GCs attenuate leptin signaling and disturb insulin signaling and reception. Ghrelin levels rise under chronic and acute stress contributing to increased food intake and weight gain. However, it is unclear if this changes are GC depended, as in patients with Cushing syndrome or under prednisolone treatment ghrelin levels are down^66,67^. Conversely, ghrelin can act on the HPA axis, increasing ACTH secretion from pituitary cells^68,69^. NPY, an orexigenic hormone, can be induced by GCs and act to promote palatable food consumption, possibly underlying the GC-driven weight gain in chronic stress. Adiponectin and GCs seem to antagonize each other, with adiponectin levels being reduced in Cushing’s patients and elevated in ADX mice, while corticosterone production is reduced by adiponectin administration^64^. Although interactions between GCs and metabolic processes are well studied, further research is needed to fully understand the complex interactions between metabolic hormones and stress responses, which could contribute to refining anti-obesity therapies.

## Sex steroids

Sex dimorphism in the circadian system arises from the diverse effects of sex hormones on physiology and behavior, which are regulated by the hypothalamic-pituitary-gonadal (HPG) axis, a central mechanism that governs reproductive processes and broader endocrine interactions^70^. The HPG axis functions through gonadotropin-releasing hormone (GnRH) neurons in the medial preoptic area, which stimulate the release of gonadotropins, luteinizing hormone (LH), and follicle-stimulating hormone (FSH). In males, LH promotes testosterone production in Leydig cells, while FSH supports spermatogenesis through Sertoli cells. In females, LH and FSH regulate ovarian function, including follicle growth, estrogen production, ovulation, and progesterone secretion by the corpus luteum^71^. A surge in LH is crucial for ovulation in females but is absent in males and after ovariectomy^72,73^. Testosterone is predominantly produced in the testes, whereas estrogen and progesterone are predominantly synthesized in the ovaries^74^. These hormones act on multiple sites in the circadian system, modifying processes at the cellular and molecular levels. Sex differences in circadian regulation include the localization of sex hormone receptors in the SCN and daily secretion rhythms^70,75,76^. Testosterone and cortisol display well-defined daily peaks in males, with testosterone declining more gradually than cortisol^77^, while estrogen and progesterone in females exhibit dynamic patterns tied to the estrous or menstrual cycle rather than depicting strong circadian rhythms^78^. These hormonal variations also influence the HPA axis, contributing to sex-specific differences in stress responses across the day^79^.

Sex hormones exert a significant tonic influence on the HPA axis, with estrogen often enhancing HPA activity. Estradiol increases stress-induced activation at all levels of the HPA axis, elevating corticotropin-releasing hormone (CRH) and AVP expression in the PVN, POMC mRNA in the pituitary, and ACTH sensitivity in the adrenal glands^80^. It also disrupts GR-mediated negative feedback on the pituitary and hypothalamus, leading to heightened stress responses^81^. Progesterone, however, can mitigate estradiol’s effects, reducing HPA activity when both hormones are present^82^. Androgens, such as testosterone, broadly suppress HPA axis activity. Gonadectomy increases stress-induced ACTH and corticosterone secretion, while testosterone replacement has the opposite effect. Testosterone’s conversion to dihydrotestosterone further enhances this inhibitory effect, reducing CRH and AVP mRNA expression in the PVN, decreasing POMC mRNA in the pituitary, and enhancing GR-mediated feedback^83^.

Estrogen plays a significant role in regulating glucose and fat metabolism by enhancing insulin sensitivity, promoting glucose uptake in peripheral tissues, and influencing lipid profiles^84^. Additionally, estrogen interacts with the hypothalamic-pituitary-thyroid (HPT) axis, modulating thyroid hormone levels, which are critical for basal metabolic rate regulation^85^. Testosterone contributes to muscle mass maintenance^86^, which indirectly influences metabolic rate and glucose utilization. These metabolic effects underscore the broader role of sex hormones in coordinating endocrine hierarchies beyond their reproductive functions, including their interaction with the HPA axis to modulate stress responses and energy balance.

There is growing evidence showing that sex hormones regulate not only reproductive but also non-reproductive processes by interacting with circadian mechanisms^75^. Sex hormones can act as rhythm drivers through their nuclear receptors acting as transcription factors to initiate gene expression. Notably, estrogen and progesterone bind to estrogen-responsive elements (*ERE*s), activating clock genes such as *Per2* and *Clock*. Sex steroid receptors are present in the SCN, suggesting they may modify circadian phase and rhythm in a sex-specific manner^87–89^. In mice, estrogen receptors are predominantly expressed in the SCN shell in females, which regulates the pace of the clock, whereas androgen receptors (AR) are more prevalent in the core in males, rich in vasoactive intestinal peptide (VIP) neurons, which regulate the phase of the clock^70,90,91^.

Recent findings suggest that estrogen modifies the SCN rhythm through astrocytes rather than neurons, specifically targeting their gap junctions and restoring rhythmicity after AVP receptor inhibition. In vitro studies show that females with high estrogen levels, resembling the proestrus phase of the estrous cycle, exhibit robust rhythmicity in the SCN, potentially mediated through astrocytic gap junctions as shown in vitro^89,92^. This implies that estrogen stabilizes the central clock’s rhythmicity, preventing it from responding to acute external cues. In contrast, ARs in the male SCN core receive direct light signals from the retinohypothalamic tract^93^, suggesting that testosterone influences the SCN phase in response to light cues. This has been confirmed with in vitro studies on male SCN, showing that simulated light exposure using NMDA on SCN explants induces distinct electrophysiological responses compared to females^94^, suggesting differential phase-shifting capabilities in both sexes.

Given these interactions, future research across species is warranted to elucidate how sex and gonadal hormones influence circadian timekeeping at cellular and molecular levels. Considering dynamic estrogen and progesterone expression across the estrous and menstrual cycles in rodents and humans, respectively, is critical. Estrogen peaks at proestrus before ovulation in females, followed by increased progesterone post-ovulation, suggesting varying effects on the circadian clock across reproductive cycle stages^75,76^. Conversely, males exhibit rhythmic testosterone expression peaking in the early morning, potentially affecting circadian rhythms throughout the day. Such sex-specific effects suggest that the hormonal modulation of HPA axis activity plays a key role in shaping differential stress responses between males and females throughout the day, potentially leading to distinct daily patterns in HPA axis output and regulation. Although the influence of sex hormones on the HPA axis is well-documented, several gaps remain. The specific mechanisms by which estradiol enhances *CRH* gene expression and disrupts GR feedback merit further investigation. Additionally, the role of non-classical androgen and estrogen receptors in modulating the HPA axis during different life stages, including puberty and menopause, is only partly understood. Future research should explore how chronic stress and metabolic conditions affect the interplay between sex hormones and the HPA axis across the lifespan.

## Thyroid hormones

Thyroid hormone (TH) synthesis is regulated by a neuroendocrine mechanism that involves the HPT axis. Parvocellular neurons release thyrotropin-releasing hormone stimulating the anterior pituitary’s release of thyroid-stimulating hormone (TSH) into the bloodstream. TSH triggers the release of THs, mainly the prohormone thyroxine (T_4_) and, to a lesser extent, the active form triiodothyronine (T_3_), by the thyroid gland. This neuroendocrine circuit is fine-tuned by the negative feedback exerted by THs on the pituitary and hypothalamus. TSH secretion shows a robust circadian regulation with an acrophase in the resting phase in humans and mice whereas the diurnal rhythms of THs are controversial as total and free THs show a shallow amplitude and often arrhythmicity^26,95–97^. Inside target cells, THs can suffer an array of reactions that may result in their activation (T_4_ conversion into T_3_) or deactivation (T_3_ into T_2_), controlled by deiodinases. Most biological effects of T_3_ are mediated through its interaction with thyroid hormone receptor α (THRα) and β (THRβ). This interaction triggers the transcription of target genes by binding TH receptors to thyroid hormone response elements (*TRE*s) in the promoter regions^98–100^. Importantly, THs are key modulators of energy metabolism, regulating glucose and lipid metabolism in various tissues^101,102^.

Although the effects of THs in regulating energy metabolism are known, whether such effects are subject to temporal regulation is a matter of investigation. We approached this question using pharmacological models to induce a low or high TH state, followed by diurnal transcriptome analysis to evaluate a possible crosstalk between THs and the circadian clock. Notably, a high TH state significantly increases energy expenditure and body temperature, affecting rhythm parameters (e.g., MESOR, amplitude, and phase) of hundreds of genes involved in glucose, lipid, cholesterol, and xenobiotic metabolism in the liver^103^. On the other hand, a low TH state reduces energy expenditure and has lesser effects on the liver circadian transcriptome^104^. Importantly, clock gene expression across different TH states is largely unaffected, suggesting that the diurnal effects of THs are downstream of the circadian clock^103,104^.

One of the concepts emerging from these observations is the role of non-rhythmic tonic signals as circadian “tuners” (or *tongeber* – German for “sound giver” in reference to the term *zeitgeber* (“time giver”) describing a phasic modulator of circadian rhythms). Since THs are largely arrhythmic, they can hardly act as rhythm drivers or *zeitgebers*. However, by interacting with intrinsic rhythmic signals changes in TH levels can affect downstream functions such as gene expression rhythms. Candidates for such intrinsic rhythms would be hormone transport (uptake), metabolization (deiodinases), and/or receptor activation (THRα and THRβ), leading to an integrated rhythmic response^26^. In line with this, the hepatocyte sensibility to T_3_ treatment is time-of-day dependent (e.g., gated) – at least in vitro^105^.

Thyroid hormones also interact with other endocrine systems such as the HPA axis. In general, increased GCs suppress the HPT axis, thus, chronically stressed mice have reduced thyroid hormone levels^106^. The effects of chronic stress on HPT axis function are not well understood, but studies generally point to a functional suppression^106,107^. In acute stress, T3 and T4 administration inhibit basal and ACTH-stimulated plasma corticosterone levels^108^. Thyroid dysfunction is discussed to affect serum levels of adipocytokines. While leptin levels are not significantly altered, hyper- but not hypothyroidism is associated with normal or increased serum concentrations of adiponectin^109^.

Taken altogether, THs are regulators of circadian energy metabolism despite them being hardly rhythmic at the hormonal level. Although the tonic metabolic effects of thyroid hormones are well-established, it is still uncertain if time of the day influences the effects of thyroid hormones. Evidence of this regulation has been observed in the liver, yet the response of thyroid hormones in other tissues, like muscle, throughout the day is still elusive. Furthermore, the exact mechanism by which a non-temporal cue triggers rhythms is still unknown, and this effect should also be tested in other tissues. Understanding the temporal regulation of thyroid hormone action could, e.g., provide benefits for the treatment of metabolic dysfunction associated steatohepatitis (MASH), a condition where thyroid hormone receptor beta agonist has shown promising effects^110^.

## Metabolic hormones

White adipose tissue (WAT) is a central metabolic organ to regulate energy homeostasis. In addition to serving as the main store of energy, it is well-known for its endocrine activity^111,112^. WAT secretes a range of cytokine-like hormones, so-called adipokines, to regulate several important functions within the tissue and act on energy functions across the whole body^113^. Leptin and adiponectin are notable examples of adipokines that regulate appetite, lipid metabolism, and fat accumulation^114,115^.

Several adipokines exhibit diurnal rhythms and their release is modulated by internal or environmental factors such as light or food intake^114^. Leptin and adiponectin show anti-phasic profiles^116^. Leptin, for example, displays a strong circadian fluctuation with the highest levels during the inactive phase and the lowest levels during the light phase in healthy humans and other diurnal mammals^14,117^. Contrary, adiponectin shows a diurnal variation with the highest levels during the day and a decrease during the late evening^116^. Since some adipokines communicate between fat tissue and the brain to regulate energy balance and other homeostatic processes, the disruption in the circadian secretion of these hormones can deteriorate human health^118,119^. Beside adipokines, other hormones are also involved in the maintenance of a healthy metabolism. Metabolic signals such as ghrelin, insulin or glucagon modulate insulin sensitivity, glucose tolerance, lipid metabolism, vascular hemostasis, and even immune responses^120,121^. Insulin is well known for its anabolic effect and its participation on fat and carbohydrate metabolism. Similar to adipokines, insulin also shows a diurnal pattern and regulates enzymes controlling triglyceride metabolism^122^. Insulin is secreted by the pancreas in response to elevated glucose levels after meals. Therefore, external cues, such as feeding patterns, have a great impact on insulin secretion. Consequently, eating during the inactive phase can lead to important changes in the circadian insulin signal^123–125^.

Extensive evidence suggests that the impairment of metabolic hormone secretion can dysregulate the clock system leading to obesity and other metabolic diseases^126,127^. Hormones can act as a *zeitgeber* modulating the clock genes and their effect on other target genes, metabolic pathways or alter the animal’s behavior. For instance, under laboratory conditions, knock-out of adiponectin results in greater food intake, altered daily profiles of lipid levels and insulin resistance in rodents^115,128^. Mice lacking functional leptin receptors present dysregulated circadian behavioral rhythms, changes in body temperature, severe overeating and altered photic responses of the SCN^129^. The alteration of the physiological endocrine signaling pathways is, frequently, the consequence of some environmental disruptor of circadian rhythms and can make a person more susceptible to health problems^14,114^.

The circadian transcription of several genes depends on the action of metabolic hormones^14^. These hormones act as circadian drivers directly regulating the expression of some genes to maintain their rhythmicity without changing clock gene expression. Adiponectin, for instance, does not affect circadian clock gene expression in some peripheral tissues, but its knockout alters the circadian rhythm of glucose and lipid metabolism^115^. In addition, adiponectin knockout decreases glucose tolerance which is associated with insulin hyposecretion in mice liver^115^. Similarly, changes in insulin receptor levels may alter dynamics of oscillation of glycemia, insulinemia and glycogen without changing food intake pattern or clock gene expression, but insulin has also been shown to act as *zeitgeber* for the liver clock^130,131^.

The efficient entrainment of endogenous rhythms to the environment is indispensable for metabolic equilibrium^132^. Dysregulation of circadian behavior such as jet lag, shift work or night eating have a substantial detrimental effect on the action of hormones that control energy balance^132,133^. A deeper understanding of the molecular mechanisms underlying circadian regulation and effects of metabolic hormones may help developing new therapeutic strategies for treating metabolic disorders. The circadian secretion and action of metabolic hormones can be impaired by the dysregulation of other endocrine systems including the action of diverse hormones like testosterone, prolactin, GCs or GH^134^. While both glucocorticoid receptors, GR and MR, regulate leptin expression, GR is the dominant receptor that is involved in cortisol-mediated regulation of adipogenesis and adipokine production^135^. While adiponectin stimulates cortisol production and steroidogenic gene expression in human adrenocortical cells, highlighting its role in regulating adrenal steroid production and metabolic activity^136^, low testosterone levels in plasma were associated with a decrease in adiponectin levels along side with the increase in parameters of the metabolic syndrome including the visceral fat, body weight, body mass index, waist circumference^137^. Contrary, testosterone is negatively correlated to leptin in plasma^138^. Increased concentration of leptin is not only associated with obesity but also effects men reproductions as it has been found that it can cause infertility^137^. This might be because a high body mass index and leptin resistance alter the Leydig cell differentiation in the testes resulting in infertility^139^. The metabolic endocrine system is complex and its association with the circadian system and other endocrine systems require further investigation. Since some sex hormones modulate the levels of important metabolic hormones, the impact of circadian dysfunction should be examined in both male and female to design novel chronobiological strategies to treat diseases such as obesity considering sex differences.

## Conclusion

The interactions between the endocrine and circadian system are complex. Many hormones show circadian modulation in their release patterns and/or in their action at target tissues. The latter can be conceptualized by direct effects on tissue physiology, resetting of local clocks, and modulation of rhythms through interaction with tissue rhythm regulation. Many hormones – such as GCs or insulin – can use more than one type of action, which allows for an even more fine-tuned circadian response. Deciphering this interaction will help in better understanding how the endocrine system modulates daily rhythms of physiology and behavior and may enable us to in a tissue-specific manner modulate hormonal action in the context of disease.

## Data Availability

No datasets were generated or analysed during the current study.
